# Supplementary figures and images for: Genome-wide analysis of transcription-coupled repair reveals novel transcription events in Caenorhabditis elegans
Source: PLoS Genet. 2024 Jul 19;20(7):e1011365. doi: 10.1371/journal.pgen.1011365 (PMC11290646; doi:10.1371/journal.pgen.1011365)

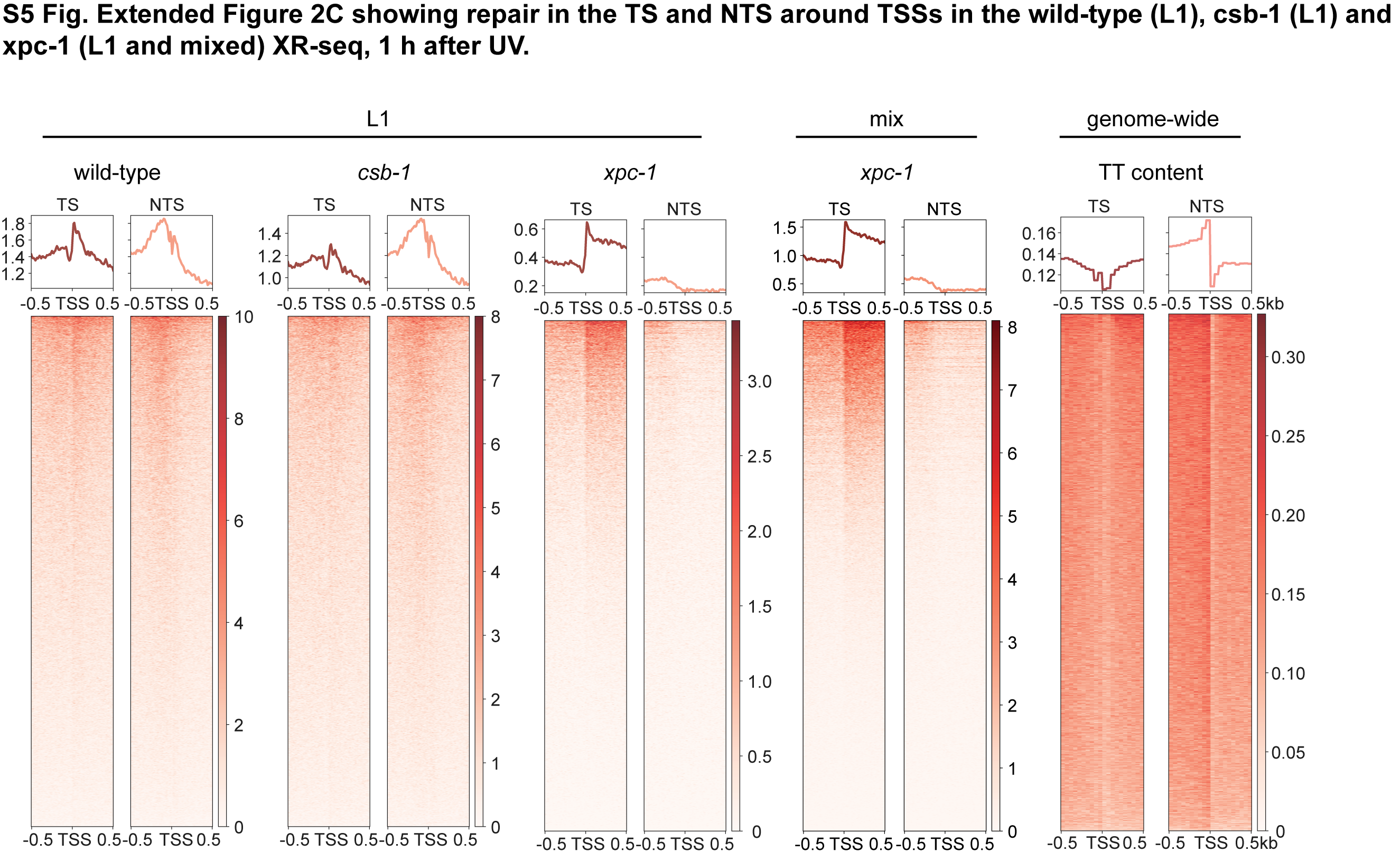

Supplement: S5 Fig — In wild-type, upstream of TSS has more repair on NTS, in contrast to more repair in TS downstream of TSS. In csb-1, despite more repair on the NTS upstream of TSS, TSS downstream repair does not show a strand preference. In xpc-1, repair in L1 worms and mixed stage worms exhibit similar profiles, proving that the repair preference in TS at TSS and its immediate downstream is not unique to the L1 worms. Near background repair at NTS versus efficient repair at TS is additional evidence of lacking global repair in xpc-1. Although profile plots (top) mask the anti-sense transcription-coupled repair upstream of TSS, a subset of TSSs exhibits upstream TCR on the non-template strand. Genome-wide TT content (right) across the same selected TSSs shows a dip in both strands at TSS. There are more TT dinucleotides on the NTS than TS upstream of TSSs, and therefore more theoretical damages which result in more repair reads in wild-type and csb-1 in that region. (TIF) [file pgen.1011365.s005.tif]

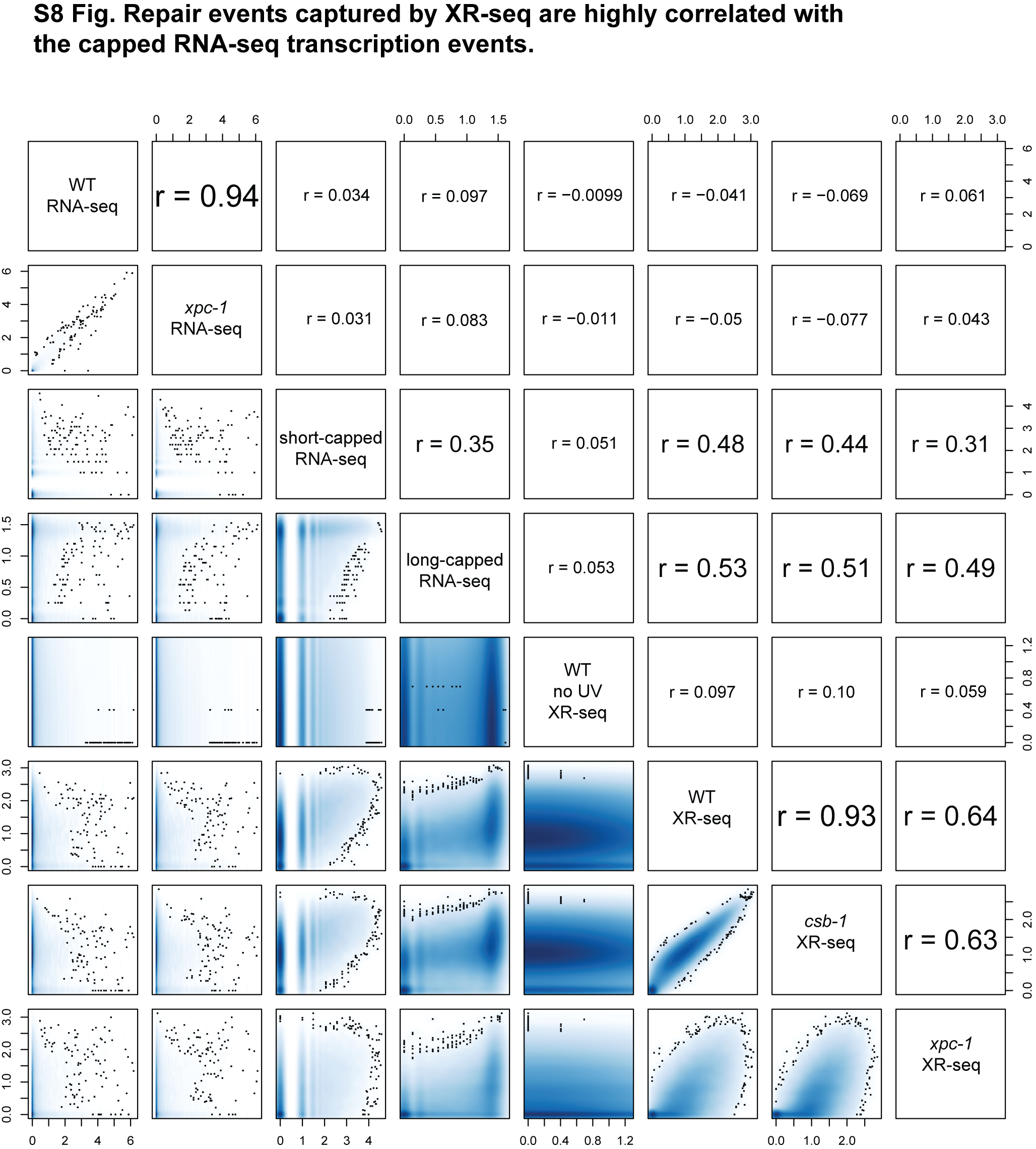

Supplement: S8 Fig — XR-seq repair signals correlate with short- and long-capped RNA-seq signals much stronger than conventional RNA-seq. Pairwise smooth scatterplots are shown on the lower triangle, where color corresponds to smoothed data density; Spearman correlation coefficients are shown on the upper triangle, with text size proportionate to the absolute value of the coefficient. Library-size-adjusted read counts from the filtered genomic bins are plotted on the original scale; XR-seq replicates were merged by taking the average, and the 1h timepoint for xpc-1 was used. (TIF) [file pgen.1011365.s008.tif]

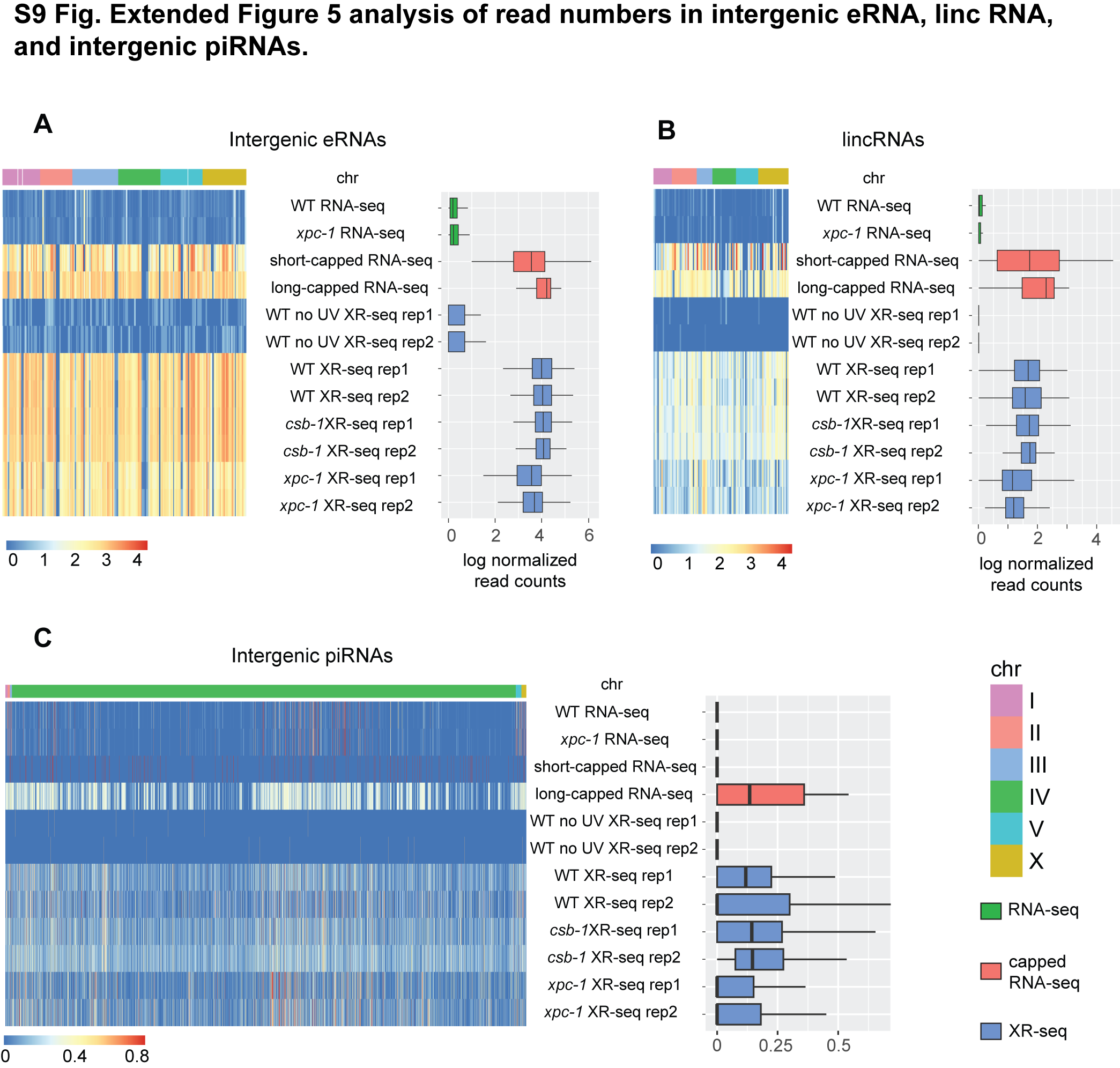

Supplement: S9 Fig — (A) Heatmaps (left) display normalized reads for intergenic enhancer RNAs (eRNAs) segregated by chromosomes. Normalization by log(x+1) was carried out, where x is library-size-adjusted read count. Bar graphs (right) represent log-normalized read counts for eRNA. Data are presented for WT and xpc-1 RNA-seq, WT long- and short-capped RNA-seq, and 2 replicates each of XR-seq from WT no UV, 1 hour after UV in WT and csb-1, and xpc-1 combined time-course (5min, 1h, 8h, 16h, 24h, and 48h). (B, C) Heatmaps and bar graphs as in A, for long intergenic non-coding RNAs (lincRNAs) and intergenic Piwi-interacting RNAs (piRNAs), respectively. (TIF) [file pgen.1011365.s009.tif]

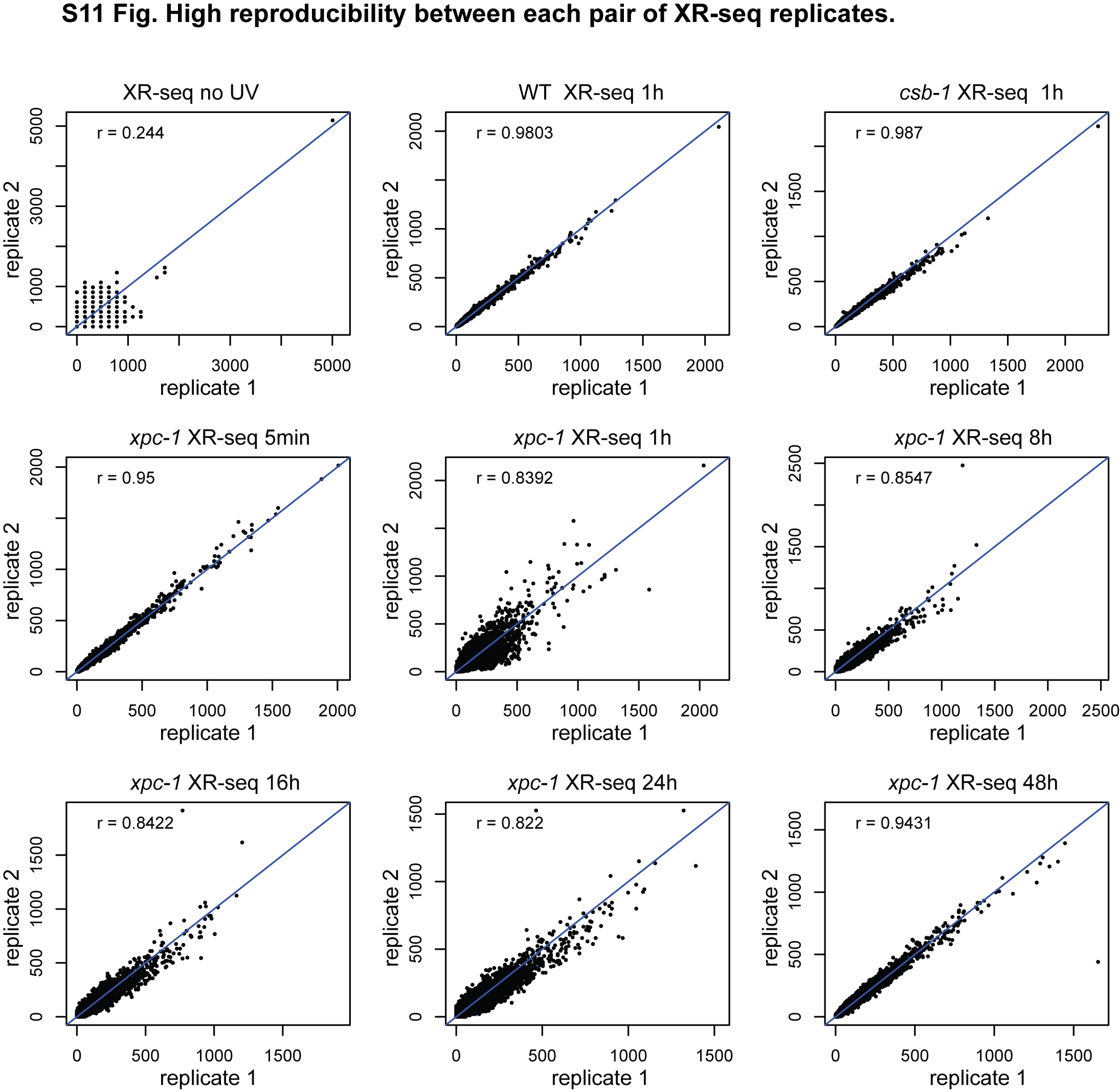

Supplement: S11 Fig — Normalized gene-specific repair is shown as each dot. Spearman correlation coefficient is shown. (TIF) [file pgen.1011365.s011.tif]
